# Top 10 research priorities for congenital diaphragmatic hernia in Australia: James Lind Alliance Priority Setting Partnership

**DOI:** 10.1136/archdischild-2024-327108

**Published:** 2024-06-16

**Authors:** Roberto Chiletti, Courtney Vodopic, Emiko Hunt, Jess Lawer, Monique Bertinetti, Stephanie Malarbi, Valerie Kyritsis, Scott Petersen, David Stewart, Jean Hellstern, Michael Stewart, Leah Hickey, David G Tingay, Trisha M Prentice

**Affiliations:** 1 The Royal Children's Hospital, Melbourne, Victoria, Australia; 2 Murdoch Children's Research Institute, Melbourne, Victoria, Australia; 3 Congenital Diaphragmatic Hernia Australia, Geelong, Victoria, Australia; 4 The University of Melbourne, Melbourne, Victoria, Australia; 5 La Trobe University, Melbourne, Victoria, Australia; 6 Monash University, Melbourne, Victoria, Australia; 7 Mater Mothers’ Hospital, South Brisbane, Queensland, Australia

**Keywords:** Intensive Care Units, Paediatric, Intensive Care Units, Neonatal, Child Development

## Abstract

**Objectives:**

The Gaps in the Congenital Diaphragmatic Hernia (CDH) Journey Priority Setting Partnership (PSP) was developed in collaboration with CDH Australia, James Lind Alliance (JLA) and the Murdoch Children’s Research Institute to identify research priorities for people with CDH, their families and healthcare workers in Australasia.

**Design:**

Research PSP in accordance with the JLA standardised methodology.

**Setting:**

Australian community and institutions caring for patients with CDH and their families.

**Patients:**

CDH survivors, families of children born with CDH (including bereaved) and healthcare professionals including critical care physicians and nurses (neonatal and paediatric), obstetric, surgical, allied health professionals (physiotherapists, speech pathologists and speech therapists) and general practitioners.

**Main outcome measure:**

Top 10 research priorities for CDH.

**Results:**

377 questions, from a community-based online survey, were categorised and collated into 50 research questions. Through a further prioritisation process, 21 questions were then discussed at a prioritisation workshop where they were ranked by 21 participants (CDH survivors, parents of children born with CDH (bereaved and not) and 11 multidisciplinary healthcare professionals) into their top 10 research priorities.

**Conclusion:**

Stakeholders’ involvement identified the top 10 CDH-related research questions, spanning from antenatal care to long-term functional outcomes, that should be prioritised for future research to maximise meaningful outcomes for people with CDH and their families.

**WHAT IS ALREADY KNOWN ON THIS TOPIC**

- Congenital diaphragmatic hernia (CDH) is a life-threatening condition requiring multicentre research collaboration to advance outcomes throughout the CDH journey.

**WHAT THIS STUDY ADDS**

- We describe the top 10 CDH-related research questions as defined through an Australian Research Priority Setting Partnership involving healthcare professionals and the CDH community.

**HOW THIS STUDY MIGHT AFFECT RESEARCH, PRACTICE OR POLICY**

- Research collaborations targeting the defined research priorities for CDH are likely to have the greatest impact for children born with CDH throughout their lifetime and their families.

## Introduction

 Congenital diaphragmatic hernia (CDH) is a life-threatening developmental defect of the diaphragm resulting in the herniation of abdominal contents into the thorax. This impacts lung and heart development, resulting in varying degrees of lung hypoplasia, pulmonary hypertension and/or left ventricle hypoplasia. Additionally, CDH is associated with other major anomalies in 30% of cases including cardiac and gastrointestinal.^1 2^

The incidence of CDH in Australia is approximately 1:2500 pregnancies, with a reported survival rate for all cases (including antenatal deaths and terminations) averaging between 50% and 60%.^3^ Antenatal diagnosis, standardisation of perinatal and postnatal medical and surgical management, protective ventilation strategies, advances in pulmonary hypertension and cardiac therapies and extracorporeal life-support techniques are contributing factors for improved survival over the last two decades.^4 5^ However, children with CDH experience prolonged hospitalisation and long-term morbidities, impacting their quality of life into adulthood.^6^

Despite attempts to standardise CDH management, significant differences remain, internationally.^7^ Existing international consensus guidelines are mainly based on case reviews and consensus expert opinions, reflecting the challenges in creating high-grade knowledge within high-risk, high-acuity and relatively rare critical care populations.^8 9^ The primary focus of medical research in CDH has been on short-term markers of disease, with limited focus on long-term and functional outcomes,^10^ despite as many as 87% of CDH survivors experiencing long-lasting morbidity, including pulmonary, gastrointestinal and neurodevelopmental problems.^11^,^14^ The CDH journey, therefore, requires both interventions that optimise disease-free survival in the intensive care unit (ICU) and coordinated multidisciplinary and evidence-based care from the prenatal period through adulthood to reduce long-term morbidity.

Research in the setting of rare diseases requires costly and time-consuming multicentre collaboration. Therefore, research that delivers value to the end user should be prioritised. Research priority setting partnerships (PSPs) address these challenges by ensuring research questions with the potential to have the greatest impact for patients and families are identified. The ‘Gaps in the CDH (Australia) Journey PSP’ aimed to identify, prioritise and share the needs and questions of people with CDH, their families or carers and healthcare professionals to inspire future research, influence future care and improve outcomes and the well-being of those diagnosed with CDH.

## Methods

CDH Australia (CDHA) and the Murdoch Children’s Research Institute (MCRI) collaborated on the project, supported by the James Lind Alliance (JLA), a not-for-profit association developed to bring together patients, families and clinicians into PSP.^15^ CDHA, a not-for-profit organisation with more than 1000 members in Australia and New Zealand, assists and supports families affected by CDH; conducting and funding education and research within Australia. CDHA funded this project and MCRI provided in-kind support.

**Figure 1:**
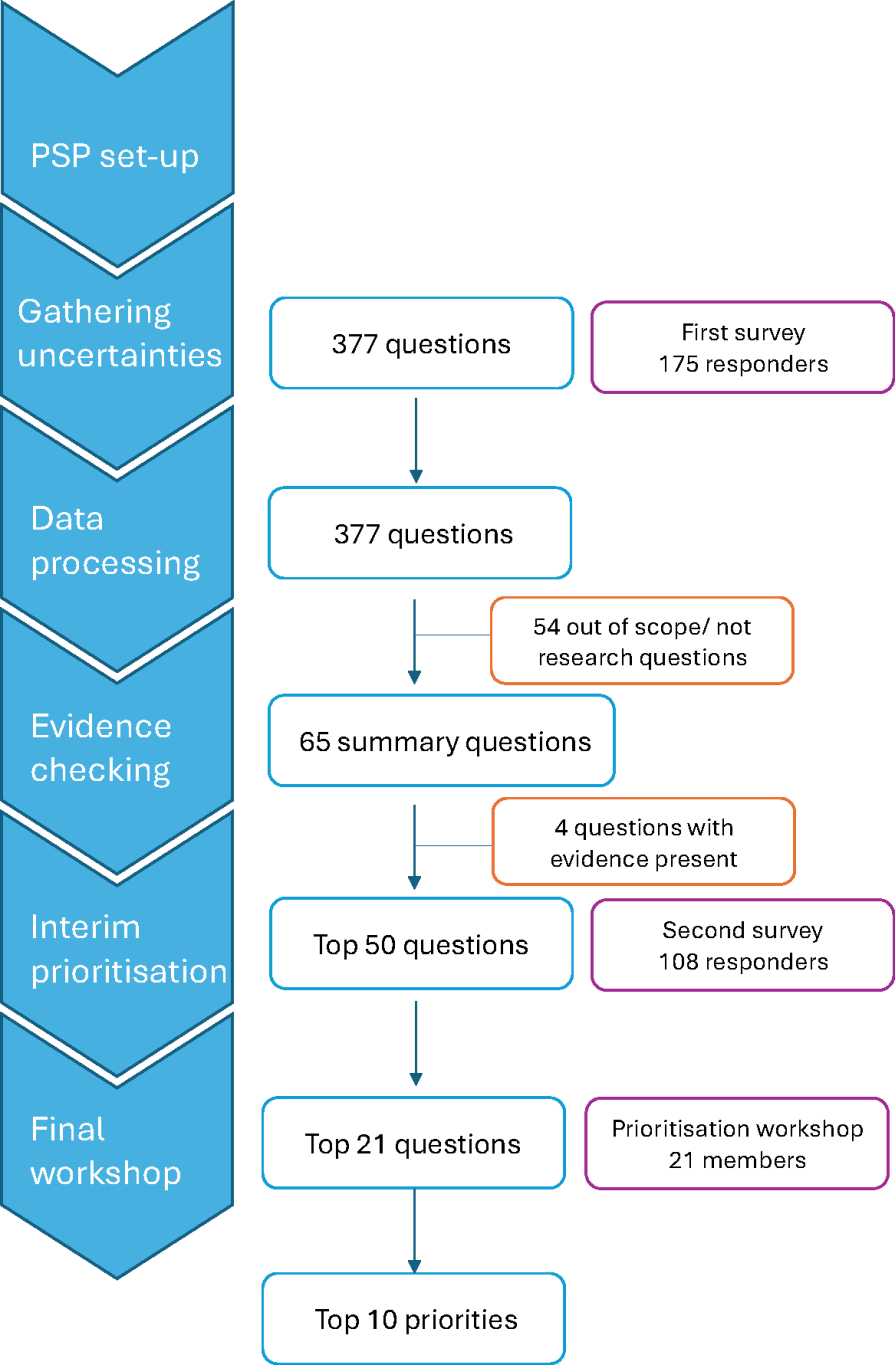
Process diagram. PSP, priority setting partnership.

Standardised JLA methodology^15^ was used, which is summarised in figure 1.

### PSP start-up (13 July 2022)

The steering group overseeing the PSP was established in July 2022 in accordance with the JLA guidelines. Its 14 members included CDHA representatives (each with lived and/or living experience of CDH) and multidisciplinary healthcare professionals (experts in the management of children affected by CDH). The CDHA Board invited families with lived or living experience to represent the CDH community. The steering group was led by a neonatologist (TMP) with expertise in managing newborns with CDH and qualitative research. A JLA adviser (Tamara Rader) supported the process in alignment with the values of the JLA, providing fair and transparent processes, and fostering respectful and balanced conversations. The MCRI assigned a project coordinator (JH) for organisational support. Meetings were held virtually to ensure broad representation.

### PSP scope

The steering group was responsible for all aspects of the PSP design and scope. The scope included:

- Management of pregnancies where CDH has been antenatally detected.
- Counselling and communication to families about management of CDH.
- Postnatal management, including resuscitation, stabilisation, surgical intervention and postoperative management.
- Longer-term follow-up and management of patients with CDH; care of expectant parents previously diagnosed with CDH.
- Improving the well-being of patients and families who have been directly impacted by a diagnosis or care of a child with CDH.

The PSP limited the scope to services relevant to Australian practice.

### Setting partnerships

Research stakeholders were identified by mapping out the CDH journey for children affected by CDH and their families, spanning the fetal–maternal unit, perinatal management, neonatal hospital care and long-term follow-up services. A diverse cultural and sociodemographic voice was prioritised and proactively sought, including review by RCH Wadja Aboriginal Family services. All steering group members contributed to the design and refinement of the first survey, including inviting members from their relevant communities to pilot the survey, with feedback incorporated prior to distribution.

### Distribution of first survey (13 November 2022)

The first survey aimed to capture raw unanswered questions. Respondents could ask up to five open questions relevant to the CDH journey and were asked to provide demographic data, including connection to CDH (see online supplemental file A).

The survey was built on the REDCap^16^ web-based research data capture platform. The survey was launched on CDHA Sunflower Sunday (13 November 2022), an annual event hosted by CDHA and promoted with links via CDHA’s and MCRI’s websites and social media channels. It was also shared through medical institutions across Australia, Australian medical colleges and research institutions, appropriate email lists, social media channels and at national conferences. The survey closed on 31 March 2023.

### Identifying unanswered questions

Questions submitted via the first survey were categorised into themes by members of the steering group. Small working groups of 2–3 steering group members were assigned a theme and collated questions into summary questions using non-technical language. Questions were excluded if answers existed within a Cochrane review or systematic review of randomised control trials (RCTs), or considered partially answered if a high-quality RCT or multicentre cohort study was completed. An electronic database search was performed of the Cochrane Database of Systematic Reviews, Ovid Medline and Embase using combinations and variations of predefined keywords. The results from the databases were merged and duplicates were removed. Only papers with full-text, published in English in the last 10 years were included for pragmatic review of whether sufficient uncertainty remained regarding the proposed research questions as per the JLA guidelines. Existing international guidelines were not considered sufficient to exclude a question. The remaining 50 summary questions were carried forward for the following prioritisation stage.

### Distribution of the second survey (19 June 2023)

The second REDCap survey was advertised and distributed through the same channels as the first survey and directly to participants from the first survey who requested contact. Participants were asked to choose and rank their top 10 CDH-related research questions from the list of 50. Recipients also provided demographic data. Participants were randomised by computer allocation to receive one of three randomly ordered question lists to reduce bias from survey fatigue. The survey remained open for 1 month. Responses were listed and categorised in order, based on the number of overall preferences and relative ranking, with subanalysis reviewing differences between preferences for the lived experience community versus healthcare professionals. Following review by the steering group, the top 21 questions were brought forward to the final workshop for discussion.

### Final workshop and top 10 questions

The workshop was held online and divided into two sessions on 2 consecutive days (2 and 3 August 2023). An expression of interest to participate in the workshop was distributed with additional selective targeting of under-represented stakeholder groups within the second survey. A total of 25 voting participants (and a support person) agreed to participate. Ultimately, two healthcare professionals (representing anaesthetic and obstetric maternal–fetal medicine) and three persons with lived experience withdrew due to personal reasons. Of the 21 who attended, 11 were healthcare professionals and 10 CDH community members (table 1). Each participant received, via post and email, a prereading package containing information on the workshop process and the list of 21 questions to be ranked in order of priority.

**Table 1:** Workshop participants

Member 1	Raising a child with CDH	VIC	Regional
Member 2	Bereaved mother	QLD	Regional
Member 3	Bereaved mother	VIC	Regional
Member 4	Raising a child with CDH	NSW	Metro
Member 5	CDH survivor	NSW	Metro
Member 6*	Raising a child with CDH	WA	Metro
Member 7	CDH survivor supported by mother (member 26)	QLD	Regional
Member 8	Raising a child with CDH	VIC	Regional
Member 9*	Bereaved mother	WA	Metro
Member 10	Bereaved mother	VIC	Metro
Member 11*	Raising two children with CDH	QLD	Regional
Member 12	Raising a child with CDH	NSW	Metro
Member 13	Clinical nurse consultant	QLD	Metro
Member 14	Neonatologist	QLD	Metro
Member 15	Neonatal clinical nurse	QLD	Metro
Member 16	Physiotherapist	QLD	Metro
Member 17	Transport NETS WA	WA	Regional
Member 18	Paediatric surgeon	QLD	Metro
Member 19†	Paediatric anaesthetist	VIC	Metro
Member 20	Senior paediatric dietician	VIC	Metro
Member 21	PICU/ECMO	VIC	Metro
Member 22	Respiratory paediatrician	VIC	Metro
Member 23	Respiratory paediatrician	NSW	Metro
Member 24	Midwife	NSW	Metro
Member 25‡	Obstetric MFM	QLD	Regional
Member 26	Clinical nurse and mother of CDH survivor (attended in support capacity to member 7)	QLD	Regional

* CDH-affected individuals/families who could not attend on the day.

† Invited anaesthetic medical representatives who could not attend on the day.

‡ Invited Oobstetric, Mmaternal F–fetal Mmedicine who could not attend on the day.

CDHcongenital diaphragmatic herniaECMOextracorporeal membrane oxygenationMFMmaternal-fetal medicineNETSnewborn emergency transport serviceNSWNew South WalesPICUpaediatric intensive care unitQLDQueenslandVICVictoriaWAWestern Australia

During the first workshop session, 4 evenly represented subgroups discussed the 21 questions and collaboratively proposed a new agreed-upon priority order, with assistance from an experienced JLA facilitator.

The priority order lists were integrated and an aggregated ranked list of questions was generated for the second session. During this session, four different subgroups were asked to discuss the aggregated list of questions from the plenary session to allow for new perspectives and final prioritisation. The final top 10 CDH research priorities in Australia were established from the aggregated subgroup lists (see table 2). As per the JLA processes, participants were surveyed on their experience of participation.

**Table 2:** Final top 10 questions and subgroup (G1–G4) ranking

Rank	Question	G1	G2	G3	G4
1	How can we optimise the neurodevelopmental outcomes of survivors of CDH?	1	1	1	1
2	How does CDH impact feeding and gut health and how can these outcomes be improved?	2	2	2	2
3	What follow-up and surveillance should patients with CDH receive (through childhood and adulthood)?	6	3	3	3
4	How can we best support the immediate care (transition) of babies born with CDH?	4	6	6	5
5	Which infants with CDH would benefit from ECMO and what is the optimal timing?	9	5	5	4
6	What are the best strategies for managing pulmonary hypertension in CDH and when should they be used?	3	8	7	7
7	What are the predictors of long-term outcomes in patients with CDH?	10	4	4	8
8	How can we best support lung growth and function of patients with CDH during their initial hospital admission?	7	7	9	6
9	What (if any) antenatal interventions improve outcomes?	5	10	8	10
10	What are the long-term respiratory outcomes of CDH?	11	12	11	11
11	How does CDH affect the growth and development of other organs before and after birth?	12	13	12	9
12	How do we best support the mental health and well-being of parents and survivors of CDH?	8	9	16	18
13	How do the lungs of babies with CDH grow and develop with time?	14	14	13	13
14	Can stem cells improve the growth of the underdeveloped or affected lung in CDH?	17	11	10	17
15	What are the best antenatal predictors of outcome in CDH and how are they best measured (eg, MRI, ultrasound)?	13	19	19	12
16	What is the best way to support the breathing of babies born with CDH before surgical repair?	16	17	15	16
17	How should follow-up care be best coordinated for patients with CDH?	15	15	14	21
18	How should labour and delivery best be supported for babies with CDH?	18	16	17	14
19	What causes or worsens CDH antenatally?	19	18	18	15
20	How can we optimise sedation and pain relief throughout the patient’s admission?	20	20	20	19
21	Does choice of inotropic medication to support blood pressure affect outcome for patients with CDH?	21	21	21	20

G1–G4=sub-groups 1–4.

Blue represents top 10 responses, yellow represents next 11–16 responses and red represents 17–21 responses.

CDHcongenital diaphragmatic herniaECMOextracorporeal membrane oxygenation

## Results

### First survey

377 questions were submitted by 175 respondents: 39.4% submitted 1 question, 20.6% 2, 12.6% 3, 6.3% 4 and 21.1% 5 questions.

Healthcare professionals and lived experience respondents comprised 55.5% and 44.5%, respectively, with neonatologists representing 43.8% of the medical professionals. The demographic characteristics are summarised in table 3, with a predominance of Caucasian women aged between 25 and 60 years living in a metropolitan area.

**Table 3:** Demographic characteristic of the survey participants

N (%)	1st survey	2nd survey
Valid responses	175	108
Respondent relation		
CDH survivor	5 (3.5)	3 (3.2)
Parent/caregiver CDH survivor	46 (31.9)	23 (24.2)
Bereaved parent of CDH child	9 (6.3)	7 (7.4)
Healthcare professional	80 (55.6)	61 (64.2)
Other	4 (2.8)	1 (1.1)
Not disclosed	31	13
Healthcare provider role		
NICU nurse	14 (17.5)	14 (23)
Midwife	1 (1.3)	0
Nurse other specialty	1 (1.3)	0
NICU doctor	35 (43.8)	30 (49.2)
Surgeon	6 (7.5)	1 (1.6)
Obstetrician	8 (10)	1 (1.6)
General paediatrician	0	0
General practitioner	1 (1.3)	0
Doctor other specialty	8 (10)	6 (9.8)
Sonographer	0	0
Dietician	1 (1.3)	1 (1.6)
Speech pathologist	2 (2.6)	0
Physiotherapist	1 (1.3)	0
Occupational therapist	1 (1.3)	1 (1.6)
Social worker	0	2 (3.3)
Psychologist	1 (1.3)	2 (3.3)
Other	0	3 (4.9)
Gender		
Male	36 (25)	27 (29)
Female	103 (71.5)	64 (68.8)
Non-binary	1 (0.7)	0
Not disclosed	4 (2.8)	2 (2.2)
Age (years)		
<15	0	0
15–24	3 (2.1)	2 (2.1)
25–40	60 (41.7)	41 (43.2)
41–60	71 (49.3)	45 (47.4)
>60	6 (4.2)	5 (5.3)
Not disclosed	4 (2.8)	2 (2.1)
Ethnicity		
Aboriginal/Torres Strait Islander	1 (0.7)	1 (1.1)
African	0	0
Asian	11 (7.6)	6 (6.4)
Caucasian	112 (77.8)	83 (88.3)
Hispanic or Latino	0	0
Maori	0	0
Pacific Islander	0	0
Not disclosed	12 (8.3)	2 (2.1)
Other	8 (5.6)	2 (2.1)
Living area		
Australian Capital Territory	4 (2.8)	3 (3.2)
New South Wales	28 (19.4)	12 (12.6)
Northern Territory	0	0
South Australia	3 (2.1)	2 (2.1)
Tasmania	2 (1.4)	0
Queensland	21 (14.6)	15 (15.8)
Victoria	57 (39.6)	50 (52.6)
Western Australia	14 (9.7)	5 (5.3)
New Zealand	10 (6.9)	5 (5.3)
Other	5 (3.5)	3 (3.2)
Living setting		
Metropolitan	108 (75)	72 (75.8)
Regional city	30 (20.8)	18 (19.8)
Remote	6 (4.2)	5 (5.3)

CDHcongenital diaphragmatic herniaNICUneonatal intensive care unit

### Interim prioritisation

From the original 377 questions 54 were excluded for being out of scope or unanswerable (eg, comments specific to an individual, or non-CDH specific). The remaining 317 questions were ordered into 8 categories: pathophysiology (16), antenatal (76), resuscitation (13), admission (75), follow-up (89), well-being (14), next-generation (6) and implementation service provision (28).

Research questions with similar content were combined to form 65 summary questions. These were checked against 2446 published articles on CDH and 50 unanswered questions were submitted for prioritisation through the second survey.

### Second survey

108 recipients completed the survey. Healthcare professionals represented 64.2% of the respondents, with 49.0% being neonatal physicians. As per the first survey, the predominant respondents were female, Caucasian and living in a metropolitan area. The top 21 ranked questions were taken to the final workshop.

### Final workshop

The final workshop included 21 participants from 4 states/territories in Australia including regional areas: 10 CDH community members (1 in a support capacity) and 11 healthcare professionals (table 1).

Table 2 summarises the final top 10 research priorities in CDH as determined during the workshop, together with individual subgroup ranking (groups 1–4). 14 participants (14; 64% persons with lived experience, 36% healthcare professionals) responded to a postworkshop feedback survey; all either agreed or strongly agreed that the facilitators were fair and impartial, and 91% either agreed or strongly agreed the process was fair and robust.

## Discussion

CDH remains one of the most complex conditions managed in perinatal medicine, yet this is the first report of research priorities in CDH using a partnership approach. The results exemplify the importance and validity of consumer participation in driving research. The CDHA community provided visibility to the experience of living with a condition with life-long implications to specialists who may deal with only an isolated aspect of CDH.

The majority of published research and consensus guidelines focus on critical care management of CDH, including optimal ventilatory settings and/or management of pulmonary hypertension.^7 8 17^ However, the top three questions prioritised by this PSP focused on the long-term quality of life of children with CDH. This highlights the CDH journey beyond the ICU with anticipated long-term multisystem morbidity including altered lung mechanics, scoliosis, gastro-oesophageal reflux^18^ and neurodevelopmental challenges such as risks of lower intellectual ability, motor challenges, autism spectrum disorder and attention deficit hyperactivity disorder.^12 13^ A recent review of the ‘unsolved problems in CDH follow-up’ summaries these into four main groups: identification of risk factors for longer-term morbidities, such as gastro-oesophageal reflux disease (GORD), correlation between prenatal predictors and late outcomes, neurodevelopmental and optimal surgical approaches based on patient characteristics.^19^ In contrast to this PSP, this ‘problem’ list was created by clinicians alone, yet encouragingly there is considerable overlap with the CDH PSP top 10 research priorities in the prioritisation of neurodevelopmental outcomes, longer-term morbidities and early prediction of these outcomes. Though the need for long-term multidisciplinary follow-up is recognised in international guidelines and consensus statements, evidence about the best composition, frequency and duration of follow-up is lacking.^20^ Small case-loads at most institutions (15–20 babies per year) highlight the need to direct international multi-centred studies towards finding solutions to the issues of greatest priority, adjusting the scope from research on acute management to longer-term follow-up and morbidity.

Research PSPs bring together diverse stakeholders that allow for new perspectives and a shared commitment to considering what will have the greatest impact for those directly impacted by the condition. A clinician with experience in two PSPs in two different roles commented that as a patient, he ‘was attracted towards choosing support and rehabilitation priorities’ and as a healthcare professional, he ‘gravitated towards pathogenetic, diagnostic and treatment priorities’.^21^ In this PSP, for example, clinicians came to appreciate the significant negative impact of gastrointestinal complications and long-term feeding: all four subgroups ranked it independently as their number two priority, acknowledging that long-term incidence of gastro-oesophageal reflux for children with CDH remains high (up to 70%) and feeding difficulties persist beyond the first 5 years of life.^6 22^

Another priority was evidence on the best short-term and long-term management approaches to pulmonary hypertension and, correlated to it, patient candidacy and timing for extracorporeal membrane oxygenation (ECMO). Pulmonary hypertension is a well-recognised complication in CDH patients and is a major contributor to early mortality, impacts admission duration and is associated with adverse neurodevelopmental outcomes.^23^,^25^ Unlike ventilatory support, there have been few advances in effective treatments for pulmonary hypertension though most are crude and designed to manage acute pulmonary hypertensive crises. Future research is needed on the role of medium-term and long-term pulmonary hypertensive therapies, such as endothelin antagonists or prostacyclin, especially as point-of-care cardiac function monitoring has become standard practice.^17 26^ ECMO is rarely used in the management of infants born with CDH in Australia and is usually reserved as rescue therapy when pulmonary hypertensive, cardiac and high-frequency ventilation support have been maximised. ECMO timing and indication remain uncertain for clinicians and families, and variability still exists internationally on ECMO patient selection, timing of mechanical support and CDH repair while on or post-ECMO.^27^

It was observed by the facilitators that some participants prioritised questions with the most immediate results and impact; research questions perceived to be ‘novel’ but ‘blue-sky thinking’ with slow anticipated translation into clinical practice (eg, stem cell therapies) were more likely to be prioritised lower. As JLA methodology excludes participants who do not identify as a lived experience expert or clinician, it is possible that the perspectives of translational CDH researchers are not reflected in the results of this PSP. Both evidence and research prioritisation may need regular review. Questions that were less specific to CDH (eg, parental mental health) were also prioritised lower, perhaps in the hope that evidence may be readily translatable from research in other conditions.

### Limitations

Online and emailed surveys generally achieve lower response rates than face-to-face surveys and may present some initial selection bias, though diverse and representative participation was a core priority. Despite these efforts, we acknowledge that the predominance of Caucasian women participants, aged 25–60 years living in metropolitan areas, may have introduced some bias to the results.

The second survey had a high representation in the healthcare cohort from Victoria and NICU, which the steering group worked to balance in the final workshop with increased participation of interstate representatives and limited presence of neonatal staff (doctors or nurses). The high representations did not appear to affect priority congruence.

Under-representation from minority groups is a known challenge worldwide, due to mistrust, time and cost constraints, and cultural factors, such as language and communication.^28^ Furthermore, unlike the UK and the USA, Australia lacks a robust system to collect race and ethnicity data to address under-representation and promote active participation at all levels of research funding.^29^

Although this survey focused on the Australian experience, the journey of parents and children with CDH is likely similar across other specialty centres internationally. We therefore hypothesise that the priorities identified by this PSP are generally applicable to CDH populations in regions with similar healthcare models and systems.

## Conclusion

Research PSP for CDH in Australia was a robust and well-accepted process by all stakeholders in determining the top 10 research priorities for CDH. Given the high congruence across healthcare professionals, and survivors and families with lived and living experience of CDH, directing research funding and resources to address these important questions spanning from antenatal care to long-term follow-up is likely to result in the greatest impact and improved outcomes for patients and their families.

## supplementary material

10.1136/archdischild-2024-327108 online supplemental file 1

## Data Availability

Data are available up reasonable request.
