# Supplementary material for: Top 10 research priorities for congenital diaphragmatic hernia in Australia: James Lind Alliance Priority Setting Partnership
Source: Arch Dis Child Fetal Neonatal Ed. 2024 Jun 16;110(1):e327108. doi: 10.1136/archdischild-2024-327108 (PMC11672073; doi:10.1136/archdischild-2024-327108)
Supplement: online supplemental file 1 [file fetalneonatal-110-1-s001.pdf]

Gaps in the CDH Journey

Identifying research priorities to improve the outcomes of babies born with Congenital Diaphragmatic Hernia. The "Gaps in the CDH Journey" project is a collaboration between the Murdoch Children's Research Institute and CDH Australia, supported by the James Lind Alliance.

This project's vision is to improve the outcomes for children born with CDH through understanding the research priorities of people with a lived experience of Congenital Diaphragmatic Hernia in Australia. (What is CDH?)

The result will be the identification of the Top 10 Priorities for CDH Research in Australia.

The Top 10 Priorities will be published and shared with key groups such as research funders, healthcare providers, hospitals, research institutes, researchers, and the wider community.

By identifying, prioritising, and sharing the needs and questions of people with CDH, their families and healthcare providers our goal is to inspire further research, influence future care, and improve outcomes and wellbeing of those diagnosed with CDH in Australia.

This is your chance to have a direct and meaningful role in optimising the outcomes of babies diagnosed with, and those affected by CDH.

About this surveyThis online survey is the first stage of the "Gaps in the CDH Journey" project and will be used to collect CDH-related questions.

You can submit up to 5 questions relating to any aspect of the CDH journey, including diagnosis, antenatal preparation, postnatal treatment, and future implications.

All questions collected in this survey will be checked to determine if they have already been answered by existing research, which will also be shared at the conclusion of this project.

The questions not yet answered by existing research will form a second survey where those affected by CDH, caregivers, and healthcare professionals will choose and prioritise what they feel are the most important.

The outcome of this project will be the identification of the "Top 10 CDH Research Priorities".

The Top 10 Priorities will be published and shared with key groups such as research funders, healthcare providers, researchers, and the wider community.

By identifying, prioritising, and sharing the needs of those affected by CDH, our goal is to inspire further research, influence future care, and improve outcomes and wellbeing of those diagnosed with CDH in Australia.

It is anticipated this survey will take 10-15 minutes to complete.

Who can take part Families in the CDH community who have received medical care in Australia are invited to participate. Healthcare providers who work with babies and families or people affected by CDH in Australia are also encouraged to have their say.

We want to hear from you if your experience with CDH is based in Australia, and you are one of the following:

A CDH survivorA parent or caregiver to a child who has survived CDHA bereaved parent or caregiver to a child with CDHA healthcare professional who works with children, adults and/or families affected by CDH Will my answers be kept confidential?Yes, all submissions to this survey are confidential.

Your submission can be anonymous, meaning we do not know who you are, or you may choose to provide your contact details if you would like to participate in the second stage of the project or receive project updates.

By participating in this survey, you are agreeing to have your questions included in our "Gaps in the CDH Journey" project. Your questions will be analysed and may be published but will not be linked to you or any organisation or group you are affiliated with.

Any contact details collected as part of this survey will be stored confidentially and securely in accordance with the Privacy Act and deleted at the end of the project. MCRI Privacy Policy and The Privacy Act

More information Murdoch Children's Research Institute MCRI Research: Gaps in the CDH journey Email: [illegible]@mcri.edu.au

CDH Australia Email: hello@cdh.org.au

James Lind Alliance

Your questions about CDH

You may submit up to 5 CDH-related questions that you would like future CDH research to address, or that highlight gaps in healthcare and management for CDH families.

Draw on your own experience and consider what is important to you.

Some areas you may want to consider are:

CDH diagnosis Pregnancy care and preparation for the birth of a baby diagnosed with CDH Birth and postnatal care for babies diagnosed with CDH Future or ongoing physical and mental health and wellbeing implications for children born with CDH Any other questions or areas for exploration
Remember these are just examples and should not limit you. Research can cover a wide variety of subjects, so anything you think of will be of interest. This is your chance to be heard and to inform research.

Select the number of questions you would like to submit?	◯ 1<br>◯ 2<br>◯ 3<br>◯ 4<br>◯ 5<br>(Please let us know what CDH-related questions are important to you by filling in the text boxes below. You may change the number of questions you submit by clicking on the numbers.)
My first question	__________________________________
My second question	__________________________________
My third question	__________________________________
My fourth question	__________________________________
My fifth question	__________________________________

About You

Please tell us a little about yourself. We will use this information to ensure we are reaching a wide range of people and to better understand the needs of different groups.

In relation to your CDH journey, which role best describes you?

- ◯ A CDH survivor
- ◯ Parent/caregiver of a CDH survivor
- ◯ Bereaved parent/caregiver of a child with CDH
- ◯ Healthcare professional
- ◯ Other

Please specify

____

What is your primary role as a Health Care Provider?

- ◯ NICU Nurse
- ◯ Midwife
- ◯ Nurse - other speciality
- ◯ NICU Doctor
- ◯ Surgeon
- ◯ Obstetrician
- ◯ General Paediatrician
- ◯ General Practitioner
- ◯ Doctor - other speciality
- ◯ Sonographer
- ◯ Dietician
- ◯ Speech Pathologist
- ◯ Physiotherapist
- ◯ Occupational Therapist
- ◯ Social Worker
- ◯ Psychologist
- ◯ Other

Please specify

____

Do you predominantly care for adult or paediatric patients with CDH?

- ◯ Children
- ◯ Adults

What is the gender you identify with?

- ◯ Female
- ◯ Male
- ◯ Non-binary
- ◯ Prefer not to say

What is your current age?

- ◯ < 15 years
- ◯ 15 - 24 years
- ◯ 25 - 40 years
- ◯ 41 - 60 years
- ◯ 61 + years
- ◯ Prefer not to say

What ethnicity do you identify with?

- ◯ Aboriginal/Torres Strait Islander
- ◯ African (including African American)
- ◯ Asian
- ◯ Caucasian
- ◯ Hispanic or Latino
- ◯ Maori
- ◯ Pacific Islander
- ◯ Prefer not to say
- ◯ Other

Please specify

____

Where do you currently live?

- ◯ ACT and other territories
- ◯ New South Wales
- ◯ Northern Territory
- ◯ South Australia
- ◯ Tasmania
- ◯ Queensland
- ◯ Victoria
- ◯ Western Australia
- ◯ New Zealand
- ◯ Other

Please specify

____

Which best describes where you live?

- ◯ Metropolitan
- ◯ Regional City
- ◯ Remote

Stay in touch

If you would like to participate in the second stage of the project, or receive project updates, please select “Yes” below and provide your contact details.

The second stage of the “Gaps in the CDH Journey” project will take place in 2023.

Participants in the second stage of the project, will help to prioritise the questions submitted in this survey to determine the Top 10 Priorities for CDH in Australia.

The results will be available at the end of 2023.

Would you like to be involved in the next survey to put the research questions in order of importance?	◯ Yes<br>◯ No
Would you like to be kept informed on the progress of the project and the results?	◯ Yes<br>◯ No

If you answered yes to either question, please let us know your contact details. Any contact details collected as part of this survey will be stored confidentially and securely in accordance with the Privacy Act and deleted at the end of the project.

Name	__________________________
Email	__________________________
